# Organ donation in India: a big gap to fill

**DOI:** 10.1016/j.lansea.2026.100843

**Published:** 2026-08-11

**Authors:** The Lancet Regional Health – Southeast

Each August, World Organ Donation Day (Aug 13) and India's National Organ Donation Day (Aug 3) provide an opportunity to reflect on one of the country's most pressing yet overlooked public health challenges. India has made remarkable progress in organ transplantation over the last 12 years, with a recently reported four-fold increase in the number of transplantations taking place since 2013, a trend that may have been supported by the establishment of the National Organ and Tissue Transplant Organisation (NOTTO) in 2014. Yet these achievements mask a widening gap between the growing demand for organs and their availability. India is a country in demographic transition. As the population ages, and the prevalence of non-communicable disease such as chronic kidney disease, liver disease, and cardiovascular disease continues to rise across the country and southeast Asia region, organ transplantation will become an increasingly important component of health care.

In this issue of *The Lancet Regional Health – Southeast Asia*, Meshram and colleagues present a stark picture; their projections estimate a high unmet solid organ transplant need in the country by 2040, with less than 35% of demand being met in the case of kidney transplantation and less than 7% of need being met in the case of lung, liver, and heart transplantation. The shortages are felt most acutely in the paediatric population. Paediatric transplantation in the region faces a dual challenge: the scarcity of appropriately sized donor organs and limited specialist expertise. Consequently, children experience longer waiting times, increased inequity in access to care, and increased risk of dying while awaiting transplantation. The key question is whether health systems can respond in time to the predicted burden of end-stage organ failure.

Contributing to this challenge is a paradox. India's transplantation programmes continue to rely heavily on living donors (where women predominate among donors, and men predominate among recipients) and the system has developed expertise in this domain. However, this success is unlikely to meet future demand sustainably. Globally, India ranks first in living donor transplants and third in total transplants but only 68th in deceased donor transplantation. Deceased organ donation remains exceptionally low in India at fewer than one donor per million population, with deceased donors accounting for less than 20% of transplants. Nearly all deceased organ donation in the country occurs after determination of brain death. Donation after circulatory death has the potential to expand the donor pool. Although legislation permits donation after circulatory death in India, it is reported only sporadically in practice, limited by the absence of national programmes and supporting frameworks.

Although cultural beliefs and awareness may influence individual decisions, successful deceased donation depends on early donor identification, family support, rapid organ retrieval, and public trust. India, like much of the region, follows an opt-in system for organ donation, making sustained efforts to build public awareness particularly important. However, consent models alone do not determine donation rates. Global evidence suggests that high-performing donation systems also rely on strong governance, timely donor identification, effective coordination, and transparent allocation processes that minimise missed opportunities.

Structural barriers further complicate access to transplantation, with both financial means and geography playing a role. Limited public health-system capacity is filled by the private sector, which drives high out-of-pocket expenditure. This is compounded by the lack of universal health coverage and by stark inter-state disparities in facilities and clinical expertise. As a result, five states in India account for most solid-organ transplants, while deceased organ donation is most concentrated in the southern and western parts of the country. These systemic failures have fuelled illegal organ trafficking in the country, in violation of the Transplantation of Human Organs and Tissues Act (THOTA), exploiting vulnerable individuals through financial incentives or coercion, and further undermining trust in deceased organ donation.

India has established a governance framework on which further progress can be built. NOTTO, supported by State Organ and Tissue Transplant Organisations (SOTTOs), has improved coordination and transparency, though considerable variation in transplant capacity persists. Tamil Nadu's TRANSTAN programme demonstrates what sustained investment can achieve through proactive donor identification, efficient retrieval networks, transparent allocation, and public–private collaboration, resulting in some of the country's highest deceased donation rates. Recent Supreme Court directives to standardise organ allocation and strengthen brain-stem death certification may further support these efforts.

Closing the gap between organ demand and supply will require a shift in perspective. Deceased organ donation should be viewed not simply as an issue of donor registration, but as a health-system function requiring investment across the donation pathway. As demand grows, deceased organ donation must be recognised as a health-system priority to strengthen public trust and realise its full potential as an act of public solidarity.

